# Review Essay: Guts, Nutrition, and History

**DOI:** 10.1093/jhmas/jraf024

**Published:** 2025-11-27

**Authors:** Ian Miller

**Affiliations:** Ulster University, Northern Ireland

**Keywords:** Guts, Digestion, Nutrition, Food history, Modernity

In recent decades, a growing number of historians have proven fascinated with the relation among food, medicine, and health, plotting out intricate histories of the sociocultural, emotional, and political aspects of nutrition, digestion, and consumption. By unravelling the complexities of eating (a matter not quite as mundane as it first appears), these historians have complemented traditional approaches by engaging with new methodologies, including those relating to the body and emotions.

Food and health have always been closely intertwined, but historically and currently that relationship is one of remarkable flux. When I published a book on the history of the stomach back in 2011, little did I foresee that the microbiome—the hidden world of microorganisms dwelling in our guts that help regulate our physical and mental well-being—was about to be elevated into a core health concept.^1^ Nor did I imagine, while writing a further book that, in part, explored the role of nutritional science in driving dietary change in post-Famine Ireland, that inadequate access to a healthy diet (“food poverty,” to use the modern-day term) was to rearise in that country during the post-COVID economic crisis.^2^

Given the timeliness and persistent importance of matters pertaining to dietary health, it seems astonishing that their history remains somewhat sidelined in academic history writing. Perhaps the study of food appears too mundane, maybe too quirky, or could it be that snobbishness exists in some circles towards historiographical studies of food? My personal view is that exploring food (and its associated physiological processes) takes us to the very heart of day-to-day historical experiences with our bodies, and how societies sought to regulate them. As such, historiographical studies of food could hardly be more important and should be integral to histories of health and medicine in particular.

Fortunately, the historiographical tone has shifted in recent years, demonstrating a growing enthusiasm for embedding methodologies from medical, cultural, and emotions history, largely replacing an outdated obsession among economic historians with historical living standards debates. Traditionally, these focused overwhelmingly on the dietary changes imposed by nineteenth-century modernization and industrialization, and were immersed deeply in qualitative methods which, as Emma Griffin argues quite persuasively, limited the nature and terms of historiographical debate.^3^ Excellent recent examples of the refocused historiography include Elizabeth Williams’s *Appetite and its Discontents,* which explores changing scientific understandings of appetite over time to help us better understand present-day health modernities. More specifically, Williams traces the eighteenth-century appearance of anxieties about eating as modern sciences displaced traditional dietary knowledge of diet, insisting that science alone was the only adequate guide.^4^ A further shining example is Manon Mathias and Alison Moore’s volume, *Gut Feeling and Digestive Health in Nineteenth-Century Literature, History and Culture* which embeds the medical, cultural, and emotional while also promoting a fuller appreciation of corporeality and digestive health.^5^

Evidently, historians from varying scholarly backgrounds and perspectives are currently invested in analyzing food, diet, and digestion. The three books reviewed here use food as a means of making fuller sense of the historical transition to western modernity. This agenda drives Steven Shapin’s book, *Eating and Being: A History of Ideas about Food and Ourselves*. Shapin’s key aim is to use long-term historical changes in thinking about food as a portal for scrutinizing the very nature of modernity and the role of our bodies within it. In a nutshell, Shapin contends that dietetic thought was largely stable and homogenous since antiquity, but rapidly transformed from the nineteenth century as modernity reared its problematic head. Some modernizing physicians of the eighteenth century had attempted to rethink traditional dietetics with mathematical, mechanical, and chemical categories, but it was really in the following century that experts comprehensively considered food intake no longer in relation to constitutions or environments but instead as a technical matter of chemistry and physiology. The humble kitchen increasingly mirrored a scientific laboratory (pp. 279-280). In modern conceptions of eating, the correct intake of proteins, fats, and carbohydrates became, for nutritional scientists, a core goal of eating (p. 288). Into the twentieth century, the idea of the calorie further promoted ideas about food intake being a matter of scientific precision.

Shapin’s lengthy book has a slightly odd structure. The main focus throughout is the early modern period, with only one chapter covering the modern period itself, which feels a little anti-climactic. Nonetheless, the research is thorough, informative, and suitably challenging. Key to Shapin’s arguments is the idea that we used to be our own physicians, but not anymore. Once modernity beckoned, doctors and scientists encouraged reliance upon their expert knowledge, displacing traditional dietetic approaches rooted in personal expertise and common sense. Nonetheless, Shapin does sensibly tease out tensions between expert knowledge and the public, rightly identifying scientific authority as contested, debated, and more often than not simply ignored. Shapin also raises compelling points about the relation between food and identity, a theme omnipresent in much food history, charting, for example, how the English were historically stereotyped as avid beef eaters. According to the old ways of thinking, beef was a cold meat, suitable for the supposedly melancholic English temperament. Eating beef helped defined who the English were while offering a glimpse into their very natures (pp. 145-148). Over time though, nutritional science advocated a one-size-fits-all approach which granted less importance to personal taste and socio-cultural influences.

The crux of the problem was (and is) that people eat food, not chemical constituents. Then, as now, the healthiest foods available, devised by scientists working with qualitative data, were bland, unappealing, tasteless, and detached from popular (and meaningful) culinary traditions. Shapin’s arguments remind of Gyorgy Scrinis’s concept of “nutritionism,” which criticizes the scientist’s crude reduction food to nutrients (still a dominant approach in our appraisals of food quality) while also blaming experts for heightening our food-related anxieties.^6^

Elsa Richardson’s book, *Rumbles: A Curious History of the Gut,* suggests greater complexities relative to Shapin’s metanarrative, in particular the persistence of holistic approaches to dietary health in the modern period, currently driven by the aforementioned microbiome. Like Shapin, Richardson attempts to pursue an ambitious chronological scope, covering antiquity up to the 2020s, but really focuses (in contrast to Shapin) largely upon post-1800. She concludes that digestion (similar to nutrition), “as a bodily process, metaphor and cultural discourse has helped to make the modern self” (p. 19). Similar also to Shapin, Richardson discusses how sociocultural ideas about food have shaped collective and national identities: steak and chips for the French, roast beef (once again) for the English.

Wariness towards the currently dominant biomedical model pervade both studies, but Richardson’s book encompasses a vaster array of primary sources, interrogating how the gut-brain connection was conceptualized outside of scientific research—in literature, art, self-help, even demonology (p. 18). Importantly, Richardson emphasizes emotions. Guts, after all, are emotional organs. Sometimes they seem happy, sometimes sad. Stomachs are scary. They growl at us, angrily communicating when they require food, as Richardson reminds at the start of her compelling book (pp. 1-2). The health of guts and minds have always seemed closely interconnected but this idea, rooted in dietary thought since antiquity, withstood the shift to modernity and the rise of nutritionism, and is even enjoying a resurgence in recent times, withstanding the forces of bio-modernity.

It is hard to single out sections of *Rumbles* for praise, as the entire book is so good. One of my personal favorite sections details how nineteenth-century railways became viewed as dangerous vehicles upon which to eat on the go. Trains hurtled around at (then) unimaginable speeds, upsetting digestive systems—a metaphor for the unnaturally quickening pace of modern life. Working lives spent stooped over desks were likewise blamed for harming digestion, office life failing to compare with the vigorous outdoor life of laborers for positive health. As Richardson wryly notes, civilization was the enemy of good digestion (p. 212). The humble office clerk, crippled by chronic gastric problems and debilitating anxiety, symbolized the problem more than adequately. Capitalism and the relentless pace of city life caused gastric distress, and then had the audacity to sell cures to its victims in the form of quack medicines, tonics, and stomach pills (pp. 140-150). This is illustrative of the rich and intriguing material scattered throughout Richardson’s *Rumbles*.

Moving on to appetites of a different kind, Rachel Hope Cleves’s *Lustful Appetites: An Intimate History of Good Food and Wicked Sex* explores associations between indulging in good food and immoral sex. This discourse linking food and sex dates to antiquity, with the stomach’s appetite supposedly encouraging the other, although Cleves’s book focuses on the modern period.

The opening sections of the book explore the eighteenth-century Parisian invention of the restaurant, a symbol of that country’s desire for luxurious food. From the late eighteenth century, French cooking secularized, freeing itself from centuries of looking upon gluttony as a Christian sin. This offered up new culinary delights and eating practices, as well as (for some) a more problematic linking of good food and sex. Restaurants emblematized these changing French sensibilities. Pleasurable eating was upheld as a social good, but French restaurants soon became internationally renowned for their sex workers and private rooms (p. 14). Some strands of French social thought insisted that some women turned to sex work precisely because of their inordinate appetites for food, caring more for the free restaurant meal than the paid sex that followed (pp. 25-26).

But while French gastronomy acquired erotic connotations, British and American culinary practices went the other way, upholding a strict puritanism and plain cooking that hardly whetted bedroom appetites. Think: Kellogg’s corn flakes, Quaker oats. Not exactly foods that arouse feelings of passion. These, in Cleves’s terminology, were “moral diets” (p. 54). Puritans, contrasting themselves with the smutty French, attacked gluttony and feasting and, in doing so, took the moral high ground, both culinary and sexually.

Much of *Lustful Appetites* investigates the female authors who wrote about food in the early twentieth century, and the ongoing association of restaurants with hedonism, bohemianism, and lesbianism. From the 1930s onward, men who showed an interest in fine cooking (or cooking of any description in many households) were considered “effete” or “homosexual” (p. 169). Indeed, as Cleves traces, gourmet tastes became a significant aspect of gay male subculture in the twentieth century (p. 170). According to homophobic perspectives, “‘real men’” never ate salad—and certainly not French quiches—but the situation was not always so straightforward. Some men realized that learning to cook might endear the opposite sex. And so, it turns out that James Bond could cook a fine meal for his female dinner guests in Ian Fleming’s early novels, all forming part of his ladies’ man persona.

Combined, a key strength of these three books is their potential appeal for both scholarly and academic audiences, a testimony to mounting academic and public interest in food-related history. Shapin, Richardson, and Cleves all demonstrate that eating, seemingly a mundane act, is a complicated, messy process. As a fundamental part of our day-to-day existence, questions about how, why, what, and when we eat have in fact constantly changed, and are always rooted firmly in sociocultural, political, and emotional life. Most importantly, the three books highlight the intricate relations that formed between food and Western modernity, complementing each other by offering fresh insight into modern ways of understanding and managing our bodies (through food and the gut). For these reasons (and many more), the books are all compelling additions to the historiography of medicine and health. “Does the gut have a history?” asks Richardson (p. 12). Yes, indeed it does, and an increasingly vibrant one that, like the rumblings of a hungry stomach, should no longer be ignored.

## Footnotes

1 Ian Miller, *A Modern History of the Stomach: Medicine, Gastric Illness and British Society, 1800-1950* (London: Pickering and Chatto, 2011).

2 Ian Miller, *Reforming Food in Post-Famine Ireland: Medicine, Science and Improvement, c.1845-1922* (Manchester: Manchester University Press, 2014).

3 Emma Griffin, “Diets, Hunger and Living Standards during the British Industrial Revolution,” *Past and Present* 239 (2018): 71-111.

4 Elizabeth A. Williams, *Appetite and its Discontents: Science, Medicine and the Urge to Eat, 1750-1950* (Cambridge: University of Cambridge Press, 2020).

5 Manon Mathias and Alison M. Moore, ed., *Gut Feeling and Digestive Health in Nineteenth-Century Literature, History and Culture* (London: Palgrave, 2018).

6 Gyorgy Scrinis, *Nutritionism: The Science and Politics of Dietary Advice* (New York: Columbia University Press, 2013).

